# Correction

**Published:** 1847-03

**Authors:** 


*Correction.*—The American Society appoint several members, from year to year, to prepare essays to be read at the next annual meeting, and these papers, whether they are read or not, have generally been published *as read* before the Society. In the 1st number of vol. 7, "A Dissertation on the Preparation of a Cavity in a Tooth, Preparatory to Plugging," by W. H. Dwinelle, is published as having been read before the Society. This paper was not *read*, and of course did not come before the Society or its members till after it was published.

Our name having been connected with a peculiar practice advocated by the author, we have many times, since its appearance in the Journal, been asked whether the practice spoken of by Dr. D., was our practice under similar circumstances, or whether we would remove all soft bone, if by so doing we should probably expose the nerve of the tooth. There is evidently some misunderstanding upon this subject. If Dr. D. means, by his description, bone which is actually *decomposed*, as constituting the only covering of the nerve, I most unhesitatingly demur to his position, either in regard to its admissibility, or to being made authority in his, or in any other case, for such a practice.

A tooth, as all are aware, is made up of earth and animal matter. The earthy portion, which is mainly lime, may be wholly dissolved and removed by an acid, and still the tooth retain its form entire, while it is kept moist.

In excavating a cavity, if we leave a portion lying in immediate contact with the nerve, which has been deprived of its lime by the action of an acid, it is *not* in a condition to retain its form, after the moisture is excluded by a plug, and, although decay, strictly speaking, would not probably go on, yet there would undoubtedly be a contraction of this *cartilaginous portion* of the bone, incompatible with the safety of the tooth.

The effect would be like drying a piece of cartilage under any other circumstances—in other words, it would not occupy the same space it did before. Now, if this should happen in immediate contact with the nerve, it would, undoubtedly, give rise to trouble. If it did not reach the nerve, it could, and of course should, be removed. We are now speaking of bone which is actually decomposed. But the bone of a tooth may become *discolored*, while not the slightest *decomposition* has taken place; and should this discoloration, *without decomposition*, be present, and extend even to the nerve, we should have no hesitation in filling the tooth without removing it, in case it could not be removed without exposing the nerve. This discolored portion is frequently *as hard* as any other portion of the tooth.

We filled a tooth not a week since, where the bone had been *stained* by an amalgam plug, and this discoloration extended far beyond the *decay*, and it would have been impossible to remove the entire *discoloration* without endangering the nerve, yet we had no hesitation in filling it. In this case there was no chance for alteration of structure in any portion which was left—certainly no chance for farther decay or decomposition of the tooth. As we do not care to have our views mistaken in regard to the operation of plugging teeth, we will state that we regard the three following items actually essential to every perfect filling:

1st. That every particle of *decomposed bone* should be removed, and especially if it is in the immediate vicinity of the nerve.

2d. That foil should always be put in perfectly dry, and free from dust or any extraneous matter.

3d. That the plug should be perfectly solidified in every part, and of course entirely impermeable to fluids.

How much others may differ from us we cannot say, but we are willing this should stand as our recorded and unvarying opinion. We may, and probably shall, in a subsequent number of the Journal, give our reasons at length for entertaining these views.—*Syracuse Ed.*

